# Editorial: Geomicrobes: Life in terrestrial deep subsurface, volume II

**DOI:** 10.3389/fmicb.2023.1169127

**Published:** 2023-03-14

**Authors:** Pauliina Rajala, Malin Bomberg

**Affiliations:** VTT Technical Research Centre of Finland Ltd., Espoo, Finland

**Keywords:** geomicorbiology, subsurface microbial ecology, carbon metabolisms, geological repositories, deep biosphere, deep biosphere sampling, terrestrial subsurface

## Introduction

The deep subsurface is, including subsurface groundwater, one of the last unknown frontiers to humankind. A significant part of life on Earth resides in the deep subsurface, hiding the great potential of microbial life of which we know only a little. The extreme conditions in deep terrestrial subsurface, such as anoxia and oligotrophy, high salinity, lack of primary production, are thought to resemble those of early Earth, which makes this environment an analog for studying early life in addition to possible extraterrestrial life in ultra-extreme conditions. Furthermore, deep subsurface microorganisms offer possibilities for novel biotechnological applications.

Much new knowledge has been gained over the last decade describing the microbial communities in different terrestrial deep biospheres. The previous Research Topic (Bomberg and Ahonen, 2017) included different ways of accessing the deep terrestrial subsurface for sampling and testing and that all three domains of life (i.e. Bacteria, Archaea, and Eukaryotes) are present in the deep subsurface. It was reported that metals drive many processes of the deep biosphere and new heavy metal resistance processes were described. Reduced iron naturally occurring in rocks as well as processed carbon steel in deep groundwater are oxidized by microorganisms. It was also reported that microbial communities in biofilms or other static habitats, such as sediment, were distinct from the planktic communities, and that the biofilms colonizing a limestone cave changed with greater distance to the cave mouth. Nitrogenous compounds, such as nitrate and ammonium were used as nitrogen source in oligotrophic, anoxic groundwater, and the difficulty to distinguish between biotically and abiotically produced methane in the ultra-deep subsurface was pondered.

The present Research Topic covers the important field of geomicrobiological research of constructed deep geological sites, such as spent nuclear fuel repositories containing metals and bentonite clay (Burzan et al.). Giovannelli et al. presents a new view of sampling strategy to study ecological features over a greater geological gradient for a broad-scale analysis of the subsurface microbial ecosystem in contrast to studying very specific sites. However, laboratory incubations should not be forgotten, as shown by Nuppunen-Puputti et al. and Mandal et al., where different microbial physiologies were revealed by enrichment cultivation, which might not have been detected otherwise. The challenge now is to elucidate the activity and functional properties of the microbial communities and to connect the biological data to that of the geological to link microbial diversity to microbial functions in the very inaccessible deep terrestrial subsurface. With this Research Topic, we aimed to collect new knowledge about the roles and functions of microbial communities in the undisturbed or disturbed subsurface and called upon our colleagues to step up to the task. The deep subsurface is of interest worldwide and the present Research Topic include samplings from Asia, South America, North America, and Europe!

## What did we learn?

The deep subsurface biosphere is one of the largest microbial ecosystems on Earth, but many fundamental questions about how life exists and thrives in this habitat are still unanswered. We know only little about the biochemical processes of the deep Earth's crust, or the microbial communities interacting with biotic and abiotic factors in the isolated aquifers or the ability of the microbial communities to adapt to changing environments. This is due much to the high costs, inaccessibility, and challenges of direct sampling of deep environments and to the fact that many deep biosphere microbial populations grow very slowly or are so-called resistant to laboratory culturing techniques. To overcome the access and cost challenges, Giovannelli et al. propose a sampling approach which involves collecting a large amount of geological, geochemical, and biological data from multiple deep seeps over large spatial scales. The proposed sampling approach combined with *in situ* experiments (Burzan et al.) and long-term microcosms studies that enable us to manipulate the environmental conditions (Nuppunen-Puputti et al.; Mandal et al.) would allow us to close the gap in our current knowledge of the activity and functional properties of the microbial communities, and to connect the biological data to that of the geological to link microbial diversity to microbial functions in the very inaccessible deep terrestrial subsurface. The slow turnover time of deep biosphere microorganisms is reflected also in the studies of this Research Topic, with experimental times ranging from several months (Mandal et al.) to several years (Nuppunen-Puputti et al.; Burzan et al.).

The importance of studying both sessile and planktic microbial communities in the deep biosphere was demonstrated by Nuppunen-Puputti et al. Sessile and planktic bacterial communities were studied in microcosms containing Finnish groundwater (the Outokumpu crystalline deep subsurface) and mica schist from the same site. The microcosms were spiked with different carbon substrates, and it was shown that the microbial communities differed between microcosm types and sessile and planktic communities. Especially, sulfate-reducing bacteria were frequent occupiers of the rock surfaces. Although the sessile and planktic bacterial communities differed in their community structure, some sort of metabolic cooperation likely exists between them and with the fungal communities.

Inorganic carbon or low-molecular-weight carbon (acetate, methane, and methanol) links multiple biogeochemical pathways and thus represents an important carbon and energy source for microorganisms in the deep crystalline bedrock (Mandal et al.; Nuppunen-Puputti et al.). Mandal et al. noted a better response of rock-hosted community toward carbon dioxide vs. bicarbonate amendment. They propose that these endolithic microorganisms could show great potential for answering the fundamental questions of deep life and their exploitation in carbon dioxide capture and conversion to useful products.

A pulse of low-molecular-weight carbon compounds in the beginning of a long-term microcosm incubation supported epilithic community development on mica schist (Nuppunen-Puputti et al.). The microcosms without additional substrates developed the lowest bacterial community richness compared to acetate, methane, or methanol-amended mesocosms (Nuppunen-Puputti et al.). Methanol supported the richest bacterial and fungal communities. Especially the *Hydrogenophaga/Serpentinomonas* attached to the rock surface had a significant response to methanol.

Deep geological repositories are considered in many countries as the most promising solution for the safe and long-term disposal of hazardous wastes. The ability of deep biosphere microorganisms to influence the environmental conditions or material integrity in deep geological repositories has remained a topic of interest due to the potential impact on the long-term safety of these repositories. In many repository concepts the spent nuclear fuel is packed in metal containers immersed in bentonite clay buffer, which when fully expanded will contain an internal pressure that will hinder microbial growth and movement toward the waste containers. Burzan et al. conducted an *in situ* experiment with compacted bentonite in boreholes of a planned repository site in the Swiss Opalinus Clay rock formation. The authors observed the growth of aerobic heterotrophs instead of anaerobes in the bentonite despite the nominally anoxic conditions. The number of microbes was predicted to increase until full saturation of bentonite would be reached and will remain at stable numbers for decades. Furthermore, the main source of microorganisms in the bentonite appeared to be the bentonite itself rather than the Opalinus Clay host rock or its porewater as previously suggested (Burzan et al.). Corrosion of metallic waste container materials by anaerobic microorganisms, most commonly sulfate-reducing bacteria, originating from porewater or groundwater has long been considered a major risk factor for long-term safety. Burzan et al. didn't find direct evidence of sulfate reduction within bentonite or of microbially induced corrosion of the carbon steel, unlike previously reported in the case of the crystalline bedrock environment.

## Conclusions

There remains still much to learn about the size of the microbial communities, the genetic variation and metabolic functions in the deepest part of the biosphere despite the advancements during the past decade. Our understanding of how deep biosphere microbes react to changing environments, be it nutrient availability or constructed environment, has greatly advanced. Shifting the focus from only the planktic community to cover also the sessile community and studying the long-overlooked fungal community in addition to better-known bacterial and archaeal communities have advanced our understanding of the diversity and functionality of the deep biosphere. Advancement in sampling techniques and access to geographically and geologically diverse sites has been crucial in building new knowledge. Still, studying the deep biosphere remains a privilege of few and more accessible and innovative sampling and laboratory scale techniques are needed.

## Author contributions

Both authors listed have made a substantial, direct, and intellectual contribution to the work and approved it for publication.
